# Lactate and lactylation in sepsis: regulation of immune-metabolic crosstalk and organ injury

**DOI:** 10.3389/fimmu.2026.1900179

**Published:** 2026-08-11

**Authors:** Zhiwang Li, Wan-Jie Gu, Tao Li, Xingui Dai, Xiang-Jie Duan, Feng Yang, Hai-Yan Yin, Zhiming Zhang, Peiyu Li

**Affiliations:** 1 Department of Anesthesiology, The First People’s Hospital of Chenzhou, Medical Education Center of Jinan University, Guangzhou, China; 2 Department of Intensive Care Unit, The First Affiliated Hospital of Jinan University, Guangzhou, China; 3 Department of Emergency Medicine, The First People’s Hospital of Chenzhou, Hengyang Medical Collage, University of South China, Chenzhou, China; 4 Department of Emergency Medicine, The First People’s Hospital of Chenzhou, Hunan University of Chinese Medicine, Chenzhou, Hunan, China; 5 Department of Critical Care Medicine, The First People’s Hospital of Chenzhou, The First Clinical College, Xiangnan University, Chenzhou, China; 6 Department of Gastroenterology, The First People’s Hospital of Chenzhou, Chenzhou, China

**Keywords:** epigenetics, lactate, lysine lactylation, metabolic reprogramming, organ dysfunction, sepsis

## Abstract

Sepsis is a life−threatening organ dysfunction caused by a dysregulated host response to infection and remains a leading cause of death worldwide. Hyperlactatemia, a hallmark metabolic disorder in sepsis, has recently been recognized as an epigenetic modulator via lysine lactylation. This Review synthesizes the evolving understanding of lactate—from a prognostic biomarker to a pathogenic mediator and, most recently, to an epigenetic modulator through lysine lactylation (Kla). Sepsis induces persistent Warburg−like glycolytic reprogramming in immune and parenchymal cells, generating lactate that not only serves as a metabolic fuel but also accumulates to drive covalent histone and non−histone Kla. Rather than merely indicating tissue hypoxia, this lactate surge directly remodels transcriptional and metabolic programs via both lactyl−CoA−dependent (p300/CBP, KAT2B) and lactyl−CoA−independent (AARS1/2) lactylation pathways. We dissect the emerging regulatory network of Kla in sepsis, including validated “writers” and “erasers”, as well as potential writers and erasers awaiting validation in sepsis models, and map their cell type–specific and substrate−specific effects on acute lung injury, cardiomyopathy, acute kidney injury, and vascular dysfunction. The identical lactylation mark—exemplified by H3K18la—exhibits a context−dependent duality, being protective in macrophages yet pathogenic in alveolar or tubular epithelia. This complexity underscores the urgent need for precision−oriented therapeutic strategies. We further explore how lactate and Kla shape the immunopathological landscape of sepsis by modulating macrophage polarization, trained immunity, neutrophil extracellular trap (NET) formation, and T−cell dysfunction, and we compare these effects with the relatively more uniform immunosuppressive role of lactylation in cancer. Finally, we map the currently known landscape of both histone and non−histone Kla across the various stages of sepsis, thereby providing new avenues for mechanism−based therapies in sepsis and other inflammation−associated disorders.

## Background

Sepsis is clinically defined as life−threatening organ dysfunction caused by a dysregulated host response to infection. Globally, an estimated 48.9 million new cases occurred in 2017, accounting for 19.7% of all deaths worldwide. The pathophysiology of sepsis involves a complex interplay between excessive inflammation (the “cytokine storm”) (1), immune paralysis (1, 2), coagulopathy (3), tissue hypoxia, metabolic disorders (1), and neuroendocrine dysregulation (4). These systemic alterations can induce multiple organ dysfunctions (5), including sepsis−induced acute lung injury (ALI/ARDS) (6), acute kidney injury (AKI) (7), acute liver injury (8), sepsis−associated intestinal dysfunction (9), sepsis−associated coagulopathy (3), septic cardiomyopathy (10), and sepsis−associated encephalopathy (SAE) (11).

Elevated blood lactate level is a hallmark of sepsis and correlates tightly with disease severity and mortality (12–14). For decades, hyperlactatemia in sepsis was attributed exclusively to hypoxic anaerobic glycolysis; On this basis, lactate has been used as a biomarker for assessing tissue perfusion and prognosis (15), forming the basis of early goal−directed therapy centered on lactate clearance (16). However, recent studies have revealed an active pathological role of lactate in sepsis (17). It has been shown that under conditions without overt hypoxia, various immune cells in sepsis (e.g., macrophages, dendritic cells, T cells) undergo metabolic reprogramming toward aerobic glycolysis (the Warburg effect), thereby generating large amounts of lactate (18). This metabolic shift not only responds to hypoxia but also reflects mitochondrial dysfunction and hyperadrenergic stimulation (19). For example, in resuscitated experimental sepsis models, although enhanced aerobic metabolism and preserved mitochondrial function are observed, the mitochondrial reserve capacity declines early in sepsis and is considered a major contributor to lactate production (20). Accumulating evidence indicates that hyperlactatemia in sepsis is not merely a marker of oxygen debt but results from inflammation−associated enhanced aerobic glycolysis (Warburg−like effect) in immune cells (21). This metabolic reprogramming is essential for supporting the biosynthetic demands and effector functions of activated macrophages, dendritic cells, and T cells – a central concept in immunometabolism (22).

Beyond its role as a metabolic intermediate, lactate also acts as a signaling molecule (e.g., activating the NLRP3 inflammasome) that influences cell function (23). Critically, lactate can also serve as a substrate to directly participate in a novel protein post−translational modification – lactylation – thereby directly linking metabolic alterations to epigenetic regulation (24). Lactylation profoundly changes protein structure and function and plays a key role in the septic environment, revealing a complex feedback loop between metabolic reprogramming and lactylation (24). In sepsis, aberrant lactate accumulation drives hyperlactylation, which has been shown to participate in NLRP3 inflammasome activation, pyroptosis, and organ injury (23).

**Figure 1:**
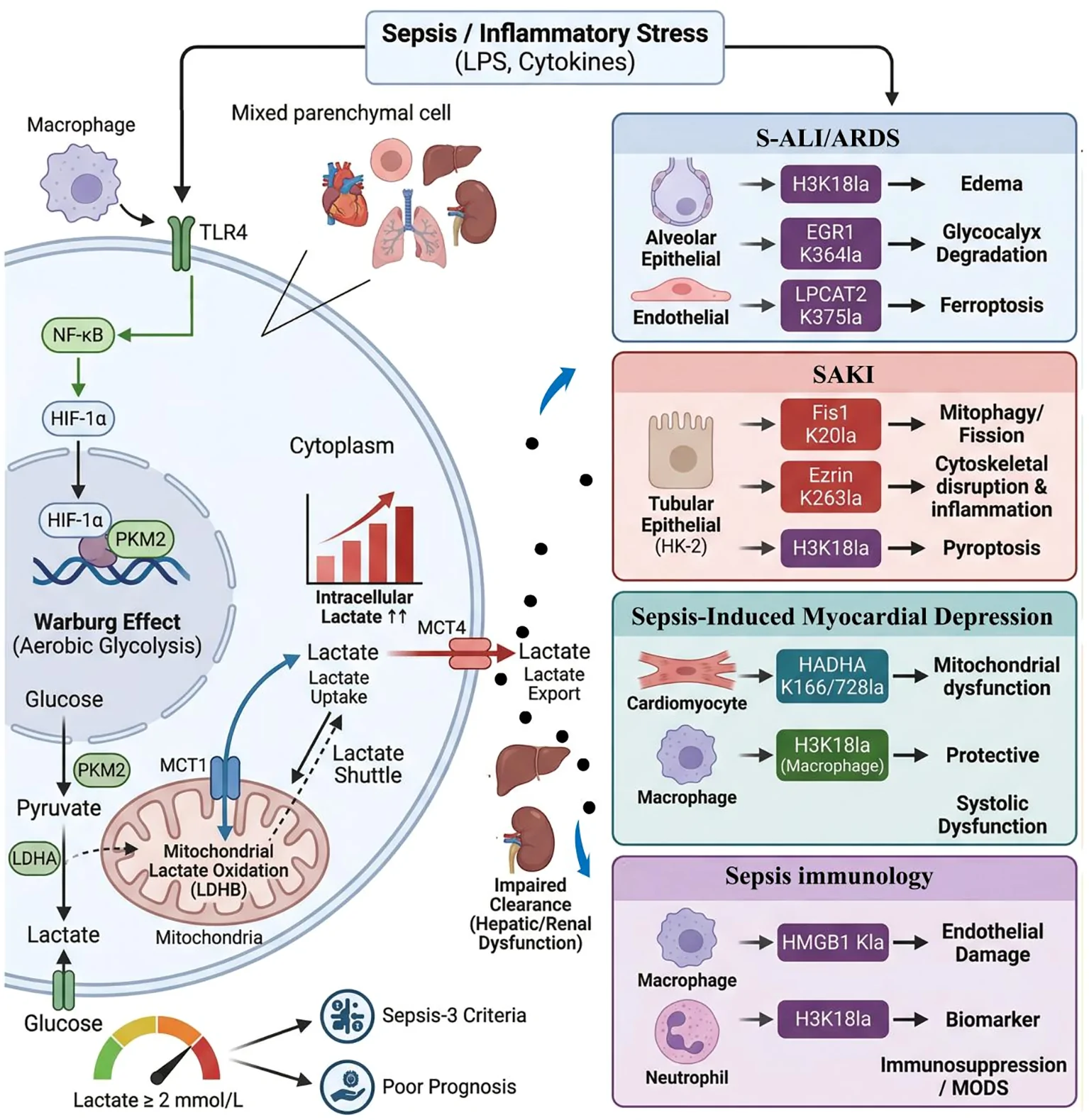
Mechanism by which altered lactate metabolism affects organ function through lysine lactylation (epigenetic modification) in sepsis.

Given the critical role of lactate in sepsis, clinicians have increasingly recognized the deleterious effects of lactate on the body. A growing number of clinical studies have focused on the impact of lactate clearance rate on the prognosis of septic patients (16, 25, 26). However, hemofiltration only transiently removes lactate from the peripheral blood and cannot eliminate lactate accumulated within immune cells. In light of the rapidly expanding body of lactate-related research, a systematic synthesis and summary are urgently needed. This review comprehensively integrates current knowledge of lactate metabolism and lactylation in sepsis, systematically delineates the molecular mechanisms by which lactate contributes to sepsis progression, and discusses therapeutic strategies targeting lactate in sepsis, thereby clarifying the potential value of lactate in clinical application and prognosis improvement (**Figure 1**). Specifically, this review aims to systematically elucidate the evolution of lactate’s role in sepsis from a “metabolic waste product” to a “pathological mediator” and further to an “epigenetic regulator”, deeply dissect the molecular mechanisms by which it exacerbates immunosuppression and mediates organ injury, and critically discuss the core regulatory network and pathological significance of lactylation as an emerging field, thereby providing a comprehensive theoretical framework for the future development of precision therapeutic strategies based on lactate metabolism and epigenetic regulation.

## Hyperlactatemia and clinical outcomes in sepsis: an updated overview

Large retrospective database studies have firmly established the association between hyperlactatemia and adverse outcomes in sepsis. In an analysis of 4,199 elderly patients from the MIMIC−IV database, each 1 mmol/L increase in lactate was associated with an OR of 1.23 (95% CI 1.18–1.28) for 28−day mortality. A non−linear relationship was observed, with a turning point at 5.7 mmol/L (27). Using the eICU database (n=10,724), in−hospital mortality increased stepwise from 13% (lactate <5 mmol/L) to 43% (5–10 mmol/L) and 79% (≥10 mmol/L); persistent elevation beyond 24 hours predicted 92% mortality (28). Importantly, trajectory analysis showed that patients with a continuously increasing lactate trajectory had a 28−day mortality of 55.6%, compared with 13.5% for those with a stable trajectory, underscoring the superior prognostic value of dynamic lactate kinetics over a single measurement (29).

Prospective cohort studies have confirmed the close association between hyperlactatemia and poor sepsis outcomes. A prospective multicenter study of 1,640 infected patients (934 with hyperlactatemia) showed that lactate level had an area under the ROC curve for in−hospital death of 0.64 (95% CI 0.61–0.72). Both moderate (4.0–9.9 mmol/L) and severe (≥10 mmol/L) hyperlactatemia were independently associated with in−hospital death (30).

Systematic reviews and meta-analyses have integrated the association between hyperlactatemia and adverse outcomes in sepsis at a higher level of evidence. A network meta-analysis including 127 studies and covering 107,445 patients showed that hyperlactatemia was significantly associated with an increased risk of sepsis mortality (OR 1.57, 95% CI 1.48–1.65), and lactate levels were significantly higher in non-survivors than in survivors (SMD 0.77, 95% CI 0.74–0.79). The prognostic value of lactate for sepsis mortality was validated by HSROC analysis, with an AUC of 0.72 (95% CI 0.68–0.76), a pooled sensitivity of 0.65, and a pooled specificity of 0.70 (31).

Even the Sepsis−3 criteria include a lactate level >2 mmol/L as one of the core diagnostic criteria for septic shock (32, 33). Collectively, these studies indicate that an elevated initial lactate level is a key quantifiable indicator for assessing sepsis severity and mortality risk. However, correlation does not equal causation. Randomized trials aimed at lowering lactate (e.g., with dichloroacetate, DCA) have not improved outcomes; indeed, Stacpoole et al. reported increased mortality at 24 hours, 72 hours, and 30 days in septic patients receiving DCA (34). Therefore, although lactate is an excellent prognostic marker, simply targeting lactate levels is unlikely to confer benefit. This paradox has fueled the search for active pathogenic roles of lactate – a role now linked to lysine lactylation.

## Metabolic sources and transport of lactate in sepsis

Under physiological conditions, lactate originates mainly from anaerobic glycolysis in skeletal muscle, brain, and erythrocytes. In sepsis, lactate production increases markedly through both hypoxia−dependent and hypoxia−independent pathways. The classic view holds that tissue hypoperfusion and microcirculatory dysfunction in septic shock lead to enhanced anaerobic glycolysis (35). This mechanism plays a major role in advanced or hypoperfusion−associated sepsis. However, studies have shown that hyperlactatemia in sepsis is often dissociated from tissue hypoperfusion, and that serum lactate levels can remain persistently elevated even after oxygen delivery has been restored or augmented, suggesting the presence of hypoxia-independent lactate production pathways (36, 37).

The concept of “aerobic glycolysis” (the Warburg effect) – initially described in tumor metabolism – has been extended to activated immune cells. LPS−stimulated macrophages exhibit markedly increased glycolytic activity (38), including enhanced glucose uptake, elevated lactate production, and accelerated extracellular acidification rate (38, 39). This metabolic shift is not passively triggered by hypoxia but is an active reprogramming of cells in response to infection and inflammation, aimed at rapid ATP generation and biosynthetic precursor supply, but it also leads to substantial lactate accumulation (40). Altered pyruvate metabolism is central to this process: as the key metabolic node connecting glycolysis to mitochondrial oxidative phosphorylation, dysregulation of pyruvate pathways directly promotes lactate generation (40). This metabolic reprogramming is finely regulated by signaling pathways such as HIF−1α and mTOR (41). Under hypoxic conditions, hypoxia−inducible factor−1α (HIF−1α) is stabilized and upregulates multiple glycolysis−related genes (e.g., HK2, PDK1), enhancing glycolytic flux (42). Notably, even in the absence of overt hypoxia in sepsis, LPS activates the NF−κB pathway through TLR4, and NF−κB family members mediate HIF−1α upregulation (43). HIF−1α induces PKM2 dimerization via the ESM1−PKM2 axis, thereby promoting the Warburg effect (44). Inflammatory signals (e.g., via the PI3K/Akt pathway) can also activate HIF−1α and induce aerobic glycolysis (45). Palsson−McDermott et al. demonstrated that PKM2 exists as a monomer/dimer in macrophages and, upon binding to Hif−1α, promotes IL−1β transcription and glycolytic metabolism (39). In addition, other pro−inflammatory cytokines, including IL−2, IL−3, IL−7, interferon−γ (IFN−γ), and tumor necrosis factor−α (TNF−α), enhance glucose metabolism and lactate production (46–48).

Besides immune cells, endothelial cells in sepsis also rely heavily on glycolysis for energy. Because endothelial cells have low mitochondrial content, their ATP generation during immune responses depends mainly on glycolysis, further exacerbating lactate accumulation (49). Meanwhile, sepsis−induced mitochondrial dysfunction and downregulation of pyruvate dehydrogenase (PDH) activity inhibit oxidative phosphorylation and the tricarboxylic acid (TCA) cycle, not only reducing ATP production but also diverting pyruvate toward lactate generation, creating a vicious cycle of increased lactate production and reduced lactate clearance (50).

Lactate is transported across cell membranes by the monocarboxylate transporter (MCT) family (notably MCT1 and MCT4) (51). MCT1 is ubiquitously expressed, has a high affinity for lactate, and mediates lactate uptake under physiological concentrations. MCT4 is induced by HIF−1α and is mainly responsible for lactate efflux from highly glycolytic cells (52). Importantly, this shuttle also occurs intracellularly: cytosolic lactate enters the mitochondria via MCT1 (or as−yet−unidentified mechanisms) on the inner mitochondrial membrane and is converted back to pyruvate by mitochondrial LDHA/B, providing a direct carbon source for the TCA cycle (53). Studies on the regulation of lactate transport by MCT1 and MCT4 in sepsis remain limited. It has been shown that MCT1 plays a key role in lactate−mediated inhibition of ZBP1−PANoptosis (54). Another study indicated that LPS downregulates MCT1 expression in mouse alveolar epithelial cells, leading to lactate transport impairment, lactate accumulation, and ultimately promoting EMT and pulmonary fibrosis (55). These findings suggest that the expression level and functional status of MCTs are central links connecting sepsis−induced metabolic disturbances with immune−inflammatory dysregulation.

## Regulation of lactate homeostasis in sepsis

Lactate clearance occurs mainly through gluconeogenesis (Cori cycle) and direct oxidation in the liver and kidneys (56–58). In sepsis, hepatic and renal dysfunction can impair lactate clearance, leading to persistent hyperlactatemia (59–63). Notably, in inflammatory macrophages, the combination of GM-CSF and IFNγ upregulates the expression of mitochondrial phosphoenolpyruvate carboxykinase (PCK2), which drives the conversion of lactate into glycogen. This process represents a compensatory metabolic adaptation that facilitates lactate clearance and sustains macrophage function within nutrient-deprived microenvironments (64). In sepsis, liver-nfiltrating monocyte-derived macrophages (MDMs) exhibit significant mitochondrial dysfunction, characterized by reduced activities of key tricarboxylic acid (TCA) cycle enzymes, including isocitrate dehydrogenase (IDH) and succinate dehydrogenase (SDH), along with impaired oxidative phosphorylation (OXPHOS), which in turn compromises their immunometabolic functions (65). This metabolic disturbance may further affect the hepatic immune microenvironment, indirectly aggravating hepatocyte injury, forming a vicious cycle of increased lactate production and decreased clearance. Clinical observations also confirm that serum lactate concentration correlates positively with SOFA/qSOFA scores, suggesting that hyperlactatemia is closely associated with the severity of organ dysfunction in sepsis (66). Downregulation of PDH activity is another important cause of lactate accumulation. PDH activity in peripheral blood mononuclear cells of septic patients is significantly reduced, and its activity correlates with patient survival. Baseline lactate level correlates negatively with PDH activity (40). Sepsis inhibits PDH activity by activating pyruvate dehydrogenase kinase (PDHK), further exacerbating lactate accumulation (67). Therefore, maintaining lactate homeostasis requires not only controlling excessive production but also preserving the clearance functions of the liver and kidneys.

## Lactate as a metabolic fuel and its metabolic diversion toward lactylation

Despite its reputation as a “metabolic waste product”, lactate is actually a preferred oxidative fuel for many tissues. Sepsis-associated hyperlactatemia has also been regarded as an adaptive phenomenon that enhances bioenergetic efficiency in the brain and heart through lactate oxidation (68). Even in the presence of glucose, lactate serves as an efficient energy source for neurons (69). Further studies have demonstrated that inhibition of the neuronal lactate transporter MCT2 reverses the depolarization (high K+)-induced enhancement of oxidative phosphorylation and the concomitant decrease in extracellular lactate, suggesting that neurons rely on MCT2-mediated lactate uptake to support oxidative metabolism under activated conditions (70). Although the above views have not been fully validated in the field of sepsis research, the collective evidence suggests that during inflammation-induced hypoglycemia or tissue hypoxia, lactate may serve as a key energy substrate for the heart, brain, and immune cells. Notably, the efficiency with which lactate serves as an oxidative substrate directly dictates its availability as a precursor for protein lactylation. Under sepsis−induced mitochondrial dysfunction, impaired lactate oxidation results in its intracellular accumulation and subsequent redirection toward nuclear lactylation pathways. This metabolic rerouting—from an oxidative fuel to an epigenetic modification substrate—constitutes a fundamental basis for the hyperlactylation state characteristic of sepsis.

## Lactylation: bridging metabolism and epigenetics in sepsis

In 2019, Zhang et al. first reported a previously unrecognized post−translational modification – lysine lactylation (Kla) – in which a lactyl group is covalently attached to lysine residues of histones (71). Using high−resolution mass spectrometry and antibodies specific for H3K18la, the researchers demonstrated that lactate, rather than other metabolites, is the direct precursor of this modification. In M1 macrophages during bacterial infection and under hypoxic conditions, elevated intracellular lactate levels strongly induce Kla. This discovery not only expands the chemical diversity of histone modifications but also directly links the glycolytic end−product lactate to epigenetic regulation, suggesting that lactate may act as a metabolic signaling molecule involved in reprogramming gene expression. To facilitate understanding of how these metabolic and epigenetic changes affect organ function in sepsis, we have summarized the mechanisms in a schematic diagram based on the literature (**Figure 1**).

## Validated and putative lactylation ‘writers’ in sepsis

In the context of sepsis, the ‘writers’ of lysine lactylation (Kla) can be divided into two categories based on whether they require lactyl−CoA as the lactyl donor. The first category comprises lactyl−CoA−dependent lactylation writers, which in sepsis include p300/CBP and KAT2B. The second category comprises lactyl−CoA−independent lactylation writers, which in sepsis include AARS1.

lactyl−CoA−dependent lactylation writers. These writers use L−lactyl−CoA as the direct substrate to catalyze lactyl group transfer to lysine residues. Central to this pathway is the conversion of L−lactate to L−lactyl−CoA by ACSS2. Subsequently, p300 utilizes L−lactyl−CoA as the L−lactyl donor to exert its lactyltransferase activity (72). Studies have confirmed that p300/CBP in septic macrophages act as lactyltransferases, catalyzing the lactylation of high−mobility group box 1 (HMGB1) (73). This modification may participate in the regulation of immune dysregulation in sepsis by altering the nucleocytoplasmic distribution or pro−inflammatory activity of HMGB1. Furthermore, in sepsis−associated acute lung injury, KAT2B has been identified as a direct lactyltransferase for the transcription factor EGR1, catalyzing its lactylation at K364 and thereby promoting EGR1 nuclear translocation and subsequent heparanase (HPSE)-mediated endothelial injury (74).

lactyl−CoA−independent lactylation writers. These writers do not rely on L−lactyl−CoA but instead catalyze lactyl transfer through other mechanisms. The most representative is the AARS1/2 family. Alanyl−tRNA synthetases (AARS1/2) normally catalyze the ligation of L−alanine to tRNA, but they have recently been identified as L−lactate sensors. Under L−lactate−rich conditions, they deviate from their canonical enzymatic activity and acquire the ability to directly bind L−lactate and catalyze ATP−dependent lactylation of lysine residues. Mechanistically, AARS1/2 first catalyze the formation of an L−lactyl−AMP intermediate, which then serves as the donor of the L−lactyl moiety to lysine residues (72). AARS1 has been identified as the key enzyme for LPCAT2 lactylation, thereby exacerbating sepsis−induced acute lung injury[ (75). These findings establish p300/CBP, KAT2B, and AARS1 as functional Kla writers in sepsis.

Other potential writers, such as members of the MYST family and aminoacyl−tRNA synthetase (ARS) family, have been primarily studied in the context of cancer, whereas the lactyltransferase activity of some of these enzymes has been validated in *in vitro* biochemical assays[(76). ATAT1, an α−tubulin acetyltransferase, has recently been shown to catalyze NAT10 lactylation, thereby affecting tRNA modification and viral replication (77). In the context of sepsis, this mechanism may be related to increased susceptibility to viral infection or abnormal recognition of self−RNA. These potential writers have not yet been functionally validated in sepsis models. Among MYST family members reported to have lactylation “writer” activity are KAT2A, TIP60 (KAT5), HBO1 (KAT7), and MOF (KAT8) (76). Among them, KAT2A acts as a lactyltransferase regulating H3K18/K14la and tumor immune evasion (78), suggesting a regulatory bridge between metabolic remodeling and immune surveillance; TIP60 (KAT5) catalyzes ALDOA−K42 lactylation, interfering with glycolysis and inhibiting valve calcification, indicating that lactylation can form a feedback loop by modifying metabolic enzymes themselves (79); HBO1 (KAT7) directly participates in H3K9la deposition at TSSs, regulating gene transcription and promoting tumorigenesis (80), and also plays a pathogenic role in H4K16la−mediated aortic aneurysm and dissection (81); MOF (KAT8) catalyzes eEF1A2−K408 lactylation to promote protein synthesis and tumorigenesis (82), and can induce PCK2−K100 lactylation to exacerbate liver ferroptosis during ischemia/reperfusion injury (83). These studies indicate that MYST family members, by recognizing site−specific substrates, are widely involved in the regulation of metabolism, inflammation, and cell death under different pathological conditions, but their specific roles in sepsis await systematic evaluation.

AARS1/2 are direct lactate transferases that catalyze lactylation independent of lactyl−CoA. This mechanistic breakthrough extends the substrate spectrum of Kla from histones to a large number of non−histone proteins, including p53, YAP, and various metabolic enzymes (76), suggesting that lactylation may act as a global metabolic signal integration mechanism, playing a regulatory role in a wide range of cellular pathways. Specifically, AARS1 can lactylate p53 and promote tumorigenesis (84), while AARS1/2 function as global lysine lactyltransferases regulating cGAS−mediated innate immunity (85). Although these findings reveal the great potential of the AARS family as lactylation writers, their specific substrates and functions in sepsis−related inflammation and immune disturbances remain unknown.

In addition, there is a controversial category of lactylation “writers”. Class I histone deacetylases (HDAC1–3) have been thought to possess both deacetylase and delactylase activities (86). However, biochemical studies have controversially suggested that class I HDACs can also catalyze the formation of lysine lactylation under certain conditions (87). This result implies a more complex writer network in which certain enzymes may possess dual “writer” and “eraser” functions. Similarly, another study found that HDAC6 can act as a lactylation “writer” at the α−tubulin−K40 site (88), further supporting the possibility that HDAC family members can have lactyltransferase activity in specific contexts. These contradictory findings indicate that the roles of HDACs in lactylation regulation require more refined subtype−, substrate−, and condition−specific analyses.

For systematic reference, we have summarized in **Table 1** the validated lactylation “writers” in sepsis, potential writers not yet validated in sepsis but with putative activity, and the currently controversial HDACs with dual activity, to provide a systematic reference for researchers working on lactylation modifications in sepsis.

**Table 1:** Validated and putative lysine lactylation ‘writers’ in sepsis.

Writer	Validated in sepsis	Specific substrate/site	Pathological context (validated/putative)
p300/CBP	Yes	H3K18la, HMGB1	Sepsis-induced endothelial dysfunction (73)
KAT2B	Yes	EGR1(K364); H3K18la	Sepsis-induced acute lung injury (74)
AARS1	Yes	LPCAT2 (K375)	Sepsis-induced acute lung injury (75)
KAT2A	No	H3K18/K14la	Tumor growth and immune evasion (78)
TIP60 (KAT5)	No	ALDOA (K42)	Glycolysis and valve calcification inhibition (79)
HBO1 (KAT7)	No	H3K9la, H4K16la	Promotes tumorigenesis (80) Aortic aneurysm/dissection (81)
MOF (KAT8)	No	eEF1A2 (K408); PCK2 (K100)	Tumorigenesis (82) Ischemia/reperfusion injury (83)
ATAT1	No	NAT10	tRNA modification and viral replication (77)
AARS1	No	p53	Colorectal cancer progression (84)
AARS2	No	PDHA1(K336); CPT2(K457/8); cGAS	Mitochondrial function & oxidative phosphorylation (89) cGAS inactivation (85)
HDAC1	Controversial	Histone H3 (e.g., K9,14,18,23,27,36,37,56,79,122)	Catalyzes lysine lactylation *in vitro* (87)
HDAC2	Controversial	Histone H3 (multiple sites as above)	HDAC2 catalyzes H3 lactylation (87)
HDAC3	Controversial	Histone H3 (presumed similar to HDAC1/2)	HDAC3/NcoR2 complex catalyzes H3 lactylation *in vitro* (87)
HDAC6	Controversial	α-tubulin (K40)	Metabolic regulation of cytoskeleton functions (88)

## Validated and putative lysine lactylation ‘erasers’ in sepsis

In the context of sepsis, four lysine lactylation (Kla) “erasers” have been clearly reported: SIRT1, SIRT3, HDAC2, and HDAC9. These delactylases regulate sepsis−associated organ injury by removing lactyl groups from specific substrates, and the same eraser can have opposite functional effects depending on the pathological context. Specifically: SIRT1 negatively regulates H3K18la levels, suggesting that it acts as an eraser for this modification and inhibits pyroptosis in renal tubular epithelial cells in sepsis−associated acute kidney injury (90). In a study on septic cardiomyopathy, SIRT1 and SIRT3 were shown to interact with HADHA and negatively regulate its lactylation at K166 and K728, thereby affecting energy metabolism in cardiomyocytes and determining the severity of septic cardiac dysfunction (91). In an exercise−induced cardioprotective model of sepsis, HDAC2 was identified as an eraser of H3K18la; knockdown of HDAC2 further increased H3K18la levels and improved cardiac function (92). This finding suggests that HDAC2−mediated delactylation of H3K18la may negatively regulate the cardioprotective effect of exercise, but its endogenous role in septic myocardial injury requires direct validation. Unlike SIRT1, which promotes pyroptosis after removing H3K18la in renal tubular epithelial cells, the role of HDAC2 in monocytes/macrophages suggests that the eraser of the same histone modification site has cell type− and pathological context−dependent effects. HDAC9 was identified as an eraser of LPCAT2 lysine 375 (K375) lactylation. In sepsis−induced acute lung injury, HDAC9 expression is downregulated, leading to increased LPCAT2−K375 lactylation, which in turn triggers ferroptosis in alveolar epithelial cells via the STAT1/SLC7A11 axis, exacerbating lung injury (75). This finding suggests that HDAC9−mediated delactylation may be protective, and its loss of function is a key factor in worsening lung injury. Collectively, these findings indicate that in sepsis, Kla erasers bidirectionally regulate organ injury and protection by acting on histone or non−histone substrates, with functions that are substrate−, cell type−, and pathological context−dependent.

Other potential Kla erasers are mainly found in the sirtuin family and HDAC family (76, 86), and their research background currently originates from fields such as cancer and cardiovascular disease, without clear functional validation in sepsis models. Although these findings are not direct evidence for sepsis, they provide important clues for future research. For example: SIRT2 has been identified as an eraser of METTL16 lysine 229 (K229) lactylation, promoting cuproptosis in gastric cancer by regulating the m^6^A modification of FDX1 (93), suggesting that SIRT2 may regulate cell death modes by connecting lactylation and RNA epitranscriptomics. Sirtuin 6 mediates H3K9 delactylation, inhibits MGMT transcription, and reverses temozolomide (TMZ) resistance in glioblastoma (GBM) (94), indicating that SIRT6 can affect DNA repair gene expression through epigenetic remodeling. HDAC1 has been identified as an eraser of SPTAN1, accelerating hepatocellular carcinoma progression, suggesting that the lactylation dynamics of cytoskeletal proteins may affect tumor invasion (95). HDAC3 acts as an eraser of H4K12 lactylation (H4K12la); inhibition of its deacylase activity leads to H4K12la accumulation and upregulation of the senescence-associated secretory phenotype (SASP), thereby aggravating vascular smooth muscle cell senescence and atherosclerosis (96), suggesting that histone delactylation can promote the senescence−associated secretory phenotype. HDAC5 has been identified as an eraser of MRE11 lysine 673 (K673) lactylation, reducing radioresistance in triple−negative breast cancer (97), suggesting that lactylation of DNA damage repair proteins may affect radiotherapy sensitivity. These studies reveal the broad potential of SIRT and HDAC family members as Kla erasers under various pathological conditions, but their specific substrates and functions in sepsis−associated immunometabolic disturbances remain to be systematically evaluated.

For systematic reference, we have summarized in **Table 2** the validated lactylation “erasers” in sepsis (SIRT1, SIRT3, HDAC2, HDAC9) and potential erasers not yet validated in sepsis (such as SIRT2, SIRT6, HDAC1, HDAC3, HDAC5, etc.). This table is intended to provide a systematic reference for researchers working on lactylation modifications in sepsis and to promote in−depth exploration of the cross−disciplinary area between metabolic disorders and epigenetic regulation.

**Table 2:** Validated and putative lysine lactylation ‘erasers’ in sepsis.

Eraser	Validated in sepsis	Modified protein	Pathological context (validated/putative)
SIRT1	Yes	H3K18la	Sepsis-associated acute kidney injury (90)
SIRT3	Yes	HADHA	Septic cardiomyopathy (91)
HDAC2	Yes	H3K18la	Septic cardiomyopathy (92)
HDAC9	Yes	LPCAT2 (K375)	Sepsis-induced acute lung injury (75)
SIRT2	No	METTL16 (K229)	Promotes cuproptosis in gastric cancer (93)
SIRT6	No	H3K9la	Glioblastoma cells (94)
HDAC1	No	SPTAN1	Accelerates hepatocellular carcinoma progression (95)
HDAC3	No	H4K12la	Atherosclerosis (96)
HDAC5	No	MRE11 (K673)	Radioresistance in triple-negative breast cancer (97)

## Landscape of protein lactylation modifications across different cell types and systems in sepsis

**Figure 2:**
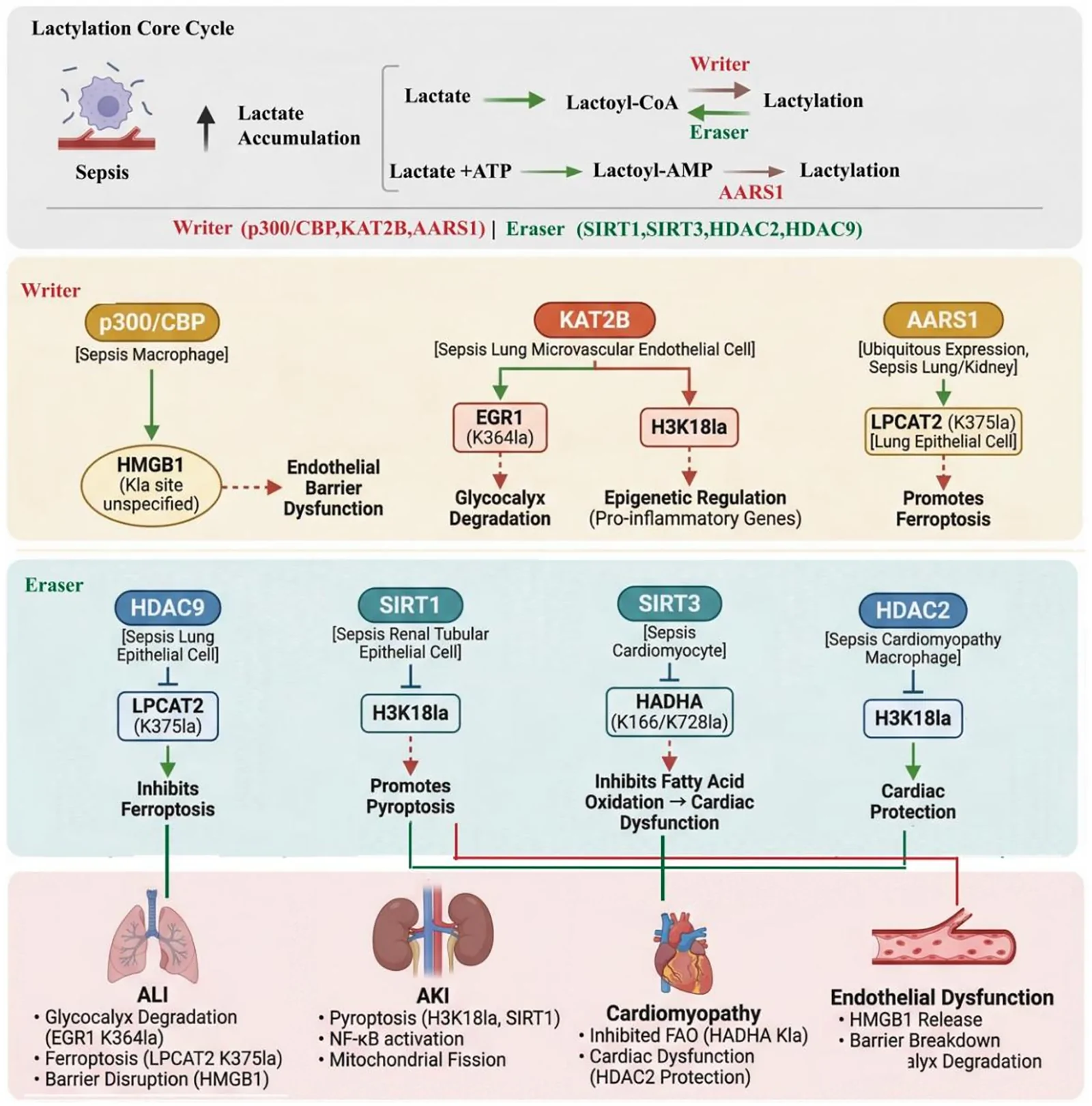
Schematic diagram of the regulatory landscape of lysine lactylation “writers”, “erasers”, and their substrates and modification sites across different systems and cell types in sepsis.

In sepsis, both histone and non−histone lactylation have emerged as key regulatory mechanisms. In different organs and cell types, lactylation modifications participate in the pathogenesis of sepsis−associated organ injury through specific substrates, exhibiting a high degree of cell type and functional dependence. To facilitate understanding of how the validated lysine lactylation “writers”, “erasers”, and related substrates and sites affect organ function in sepsis, we have drawn a schematic diagram of the protein lactylation landscape across different cell types and systems in sepsis based on the above summary (**Figure 2**).

In cells associated with acute lung injury (ALI), lactylation modifications are widely involved in barrier disruption and inflammation amplification in alveolar epithelium, pulmonary microvascular endothelium, and pulmonary vascular endothelium. Specifically: In alveolar epithelial cells, histone H3K18la exacerbates pulmonary edema (98); in lung epithelial cells, LPCAT2 K375 lactylation directly participates in septic lung injury (75). In pulmonary microvascular endothelial cells, EGR1 K364 lactylation and histone H3K18la together cause endothelial glycocalyx degradation and disrupt the vascular barrier (74). Lactate induces the lactylation of macrophage-derived cold-inducible RNA-binding protein (CIRP) and promotes its extracellular release. Once internalized by pulmonary vascular endothelial cells (PVECs) via a Toll-like receptor 4 (TLR4)-mediated endocytic pathway, extracellular CIRP (eCIRP) competitively binds to Z-DNA binding protein 1 (ZBP1), thereby disrupting the interaction between ZBP1 and tripartite motif-containing protein 32 (TRIM32), an E3 ubiquitin ligase that targets ZBP1 for proteasomal degradation. This interference stabilizes ZBP1 protein levels, which in turn potentiates ZBP1-dependent PANoptosis in PVECs and exacerbates acute lung injury (99). Meanwhile, H3K14la promotes SLC40A1/transferrin−mediated ferroptosis, driving endothelial dysfunction in sepsis−induced ARDS (100). These findings indicate that lactylation acts as a central mediator of ALI through multi−cell−type, multi−substrate synergy in the pulmonary and vascular systems.

In kidney injury, multiple lactylation events in renal tubular epithelial cells (HK−2) have been reported to aggravate sepsis−associated acute kidney injury. Among them, Fis1 K20 lactylation participates in the regulation of mitochondrial fission (101); Ezrin K263 lactylation affects cytoskeletal connections (102); and histone H3K18la similarly exacerbates kidney injury in these cells (102). In addition, HMGB1 lactylation (site unspecified) in neutrophils has been shown to aggravate sepsis−associated acute kidney injury (103). These findings indicate that lactylation can promote renal tubular injury and deterioration of renal function through multiple mechanisms, including regulation of mitochondrial dynamics, cytoskeletal integrity, epigenetics, and immune cell infiltration.

In myocardial injury, HADHA K166 and K728 lactylation in cardiomyocytes promote sepsis−induced cardiac dysfunction (91). HADHA is a key enzyme in fatty acid β−oxidation, and its lactylation inhibits energy metabolism, providing direct evidence for a metabolic failure mechanism in septic cardiomyopathy. Notably, histone H3K18la in macrophages has been reported to be protective in the same disease context, improving septic cardiomyopathy (92), suggesting that lactylation modifications can have opposite functional effects in different cell types.

At the immune cell level, HMGB1 lactylation in macrophages exacerbates sepsis−induced endothelial barrier dysfunction (73); meanwhile, histone H3K18la in peripheral blood mononuclear cells (PBMCs) can serve as a diagnostic and prognostic marker for patients with septic shock (104). Different substrates in the same cell, or the same modification site under different pathological conditions, may have both damaging and protective roles, reflecting the complex regulatory network of lactylation in immune responses. HMGB1 lactylation in neutrophils has also been reported to promote kidney injury (103), suggesting that HMGB1 lactylation may have a consistent pro−inflammatory, pro−injury effect across different immune cells.

Collectively, the above findings reveal several characteristics of lactylation modifications in sepsis. The same modification site (e.g., H3K18la) exhibits cell type specificity: it protects cardiac function in macrophages (92) but aggravates injury in alveolar epithelial cells (74)and renal tubular epithelial cells (90, 102). This may be related to differences in intrinsic transcriptional regulatory networks and metabolic microenvironments among different cells. Second, lactylation substrates show functional modularity: metabolic enzymes (e.g., HADHA, Fis1), structural proteins (e.g., Ezrin), transcription regulatory proteins (e.g., EGR1), and damage−associated molecules (e.g., HMGB1) can all be lactylated, affecting energy metabolism, mitochondrial dynamics, cytoskeletal integrity, and inflammatory signaling pathways, respectively. Third, in lung injury, lactylation events across multiple cell types act synergistically to cause disease: lactylation modifications in alveolar epithelial cells, endothelial cells, and immune cells collectively amplify the disruption of the vascular and alveolar barriers. Notably, most studies have identified specific lactylation sites, providing precise molecular targets for developing site−specific antibodies or mutagenesis tools and highlighting the potential for site−directed intervention. Future studies should further elucidate the upstream regulatory mechanisms that account for the functional differences of the same modification site in different cells and explore the crosstalk between lactylation and other post−translational modifications (e.g., phosphorylation, acetylation, ubiquitination). For convenient reference by future researchers, the validated protein lactylation modifications in different cell types in sepsis are summarized in **Table 3**.

**Table 3:** Landscape of protein lactylation modifications across different cell types and systems in sepsis.

Organ/system	Cell type	Lactylated protein/site	Functional category	Role in sepsis
Systemic/Immune	Macrophage	HMGB1 (unspecified)	DAMPs	Sepsis-induced endothelial barrier dysfunction (73)
	Macrophage	H3K18la	Histone	Diagnostic/prognostic marker for septic shock (104)
	Neutrophil	HMGB1 (unspecified)	DAMPs	Sepsis-associated acute kidney injury (103)
Septic Cardiomyopathy	Macrophage	H3K18la		Septic cardiomyopathy (92)
	Cardiomyocyte	HADHA (K166, K728)	Metabolic enzymes	Sepsis-induced cardiac dysfunction (91)
Sepsis-associated acute lung injury (S-ALI)	Alveolar epithelial cell	H3K18la	Histone	Septic pulmonary edema (98)
	Mouse Pulmonary Microvascular Endothelial Cells	EGR1(K364)	Transcriptional/epigenetic regulators	Endothelial glycocalyx degradation, disrupts vascular barrier (74)
		H3K18la	Histone	Endothelial glycocalyx degradation (74)
	Vascular Endothelial Cell (EC)	H3K14la	Histone	Endothelial ferroptosis, vascular dysfunction (100)
	Pulmonary Vascular Endothelial Cell (PVEC)	CIRP	DAMPs	Sepsis-associated acute lung injury (99)
	Lung epithelial cell	LPCAT2(K375)	Metabolic enzymes	Sepsis-induced lung injury (75)
Sepsis-associated acute kidney injury(S-AKI)	HK-2	Fis1(K20)	Metabolic enzymes	Sepsis-associated acute kidney injury (101)
		Ezrin(K263)	Cytoskeletal/structural proteins	Sepsis-associated acute kidney injury (102)
		H3K18la	Histone	Sepsis-associated acute kidney injury (102)
		H3K18la	Histone	Sepsis-associated acute kidney injury (90)
Septic Vascular Endothelial Injury	Human Umbilical Vein Endothelial cells (HUVECs)	ENO1(K71)	Metabolic enzymes	Sepsis-induced vascular endothelial dysfunction (105)

To systematize these findings, we categorize the currently reported non−histone Kla substrates into four major functional classes: (1) metabolic enzymes, including HADHA (fatty acid oxidation), Fis1 (mitochondrial fission), ENO1 (glycolysis), and LPCAT2 (phospholipid metabolism), which collectively regulate energy metabolism and metabolic flux; (2) cytoskeletal/structural proteins, represented by Ezrin (membrane−cytoskeleton linker), which influence cell morphology, polarity, and barrier integrity; (3) transcriptional/epigenetic regulators, such as EGR1 (transcription factor) and HMGB1 (nuclear function), which directly modulate inflammatory gene expression; and (4) damage−associated molecular patterns (DAMPs), exemplified by CIRP, which promotes pulmonary endothelial cell death.

To systematically dissect the functional differences of lysine lactylation (Kla) between sepsis and cancer and their therapeutic implications, we have established a cross−pathological comparative framework (**Table 4**). At the cellular level, Kla in sepsis involves a multi−cell, multi−organ interplay among immune cells (macrophages, neutrophils, T cells) and parenchymal cells (alveolar epithelia, renal tubular epithelia, cardiomyocytes), whereas Kla in cancer is largely confined to immune cells within the tumor microenvironment (TAMs, CD8^+^ T cells, Tregs, MDSCs) and tumor cells themselves, exhibiting a “local microenvironment−driven” profile. At the substrate level, sepsis involves histones (H3K18la, H3K14la), metabolic enzymes (HADHA, Fis1, LPCAT2, ENO1), cytoskeletal proteins (Ezrin), and DAMPs (HMGB1, CIRP), showing functional diversification; cancer substrates are concentrated in histones (H3K18la, H3K9la, H4K16la) and oncogenic proteins (p53, cGAS, PKM2, CD44), with a focus on proliferation–immune evasion axes. Functionally, the same modification (e.g., H3K18la) exhibits a “dual face” in sepsis—protective in macrophages (promoting Arg1−driven repair) but pathogenic in alveolar/tubular epithelia (ferroptosis/NF−κB activation)—whereas in cancer, Kla is predominantly pro−tumorigenic, driving immune suppression, metabolic reprogramming, and therapy resistance. These differences dictate fundamentally distinct therapeutic strategies: sepsis demands cell−type−selective intervention (enhancing protective Kla in immune cells while suppressing pathogenic Kla in parenchymal cells), whereas cancer favors broad Kla inhibition combined with immune checkpoint blockade to restore immune surveillance. This framework provides a clear translational roadmap for Kla−targeted drug development across disease contexts.

**Table 4:** Systematic comparison of lysine lactylation modifications between sepsis and cancer.

Comparison dimension	Sepsis	Cancer
Affected cell types	Macrophages (73, 92, 104), neutrophils (103), T cells (106), alveolar epithelial cells (74, 98), renal tubular epithelial cells (90, 101, 102), cardiomyocytes (91), vascular endothelial cells (74, 100, 105)	CD8^+^ T cells (109, 110), regulatory T cells (111), myeloid−derived suppressor cells (112), tumor cells (80, 82, 84, 89)
Specific Kla substrates involved	Histones: H3K18la (74, 90, 92, 98, 102, 104), H3K14la (100); Metabolic enzymes: HADHA (91), Fis1 (101), LPCAT2 (75), ENO1 (105); Structural protein: Ezrin (102); DAMPs: HMGB1 (73, 103), ZBP1 (99); Transcription factor: EGR1 (74)	Histones: H3K18la (78, 110, 111), H3K9la (80, 94), Signaling proteins: p53 (84), SPTAN1 (95), eEF1A2 (82), NAT10 (77), CD44 (109), MRE11 (97), METTL16 (93)
Functional outcomes	Context−dependent duality: H3K18la drives reparative polarization via Arg1 in macrophages (92); promotes ferroptosis via METTL3–m^6^A–ACSL4 in alveolar epithelia (98); activates RhoA–NF−κB in renal tubular epithelia (90, 102); HMGB1 lactylation induces NETosis and kidney injury (103); HADHA lactylation impairs fatty acid oxidation and cardiac function (91).	Predominantly pro−tumorigenic: H3K18la upregulates Nur77 to induce CD8^+^ T cell dysfunction, or upregulates TNFR2 to enhance Treg suppression (110, 111); CD44 lactylation inhibits AKT signaling and antitumor immunity (109); p53 lactylation promotes proliferation (84); H3K9la suppresses MGMT and drives chemotherapy resistance (94); MRE11 lactylation enhances radioresistance (97); H3K14la can inhibit vascular calcification (79).
Implications for therapeutic strategies	Preserve protective Kla in immune cells; Selectively block pathogenic Kla in parenchymal cells.	Broad−spectrum Kla inhibition combined with immunotherapy; General reduction of Kla to restore immune surveillance.

## Immunological consequences of lactate and Kla in sepsis

Lactate and its downstream lysine lactylation (Kla) exert profound and cell type–specific effects on various immune cell populations during sepsis. These immunological effects encompass both innate and adaptive immunity, involving macrophage polarization, trained immunity, neutrophil dysfunction, and T-cell impairment, which collectively shape the disease trajectory from hyperinflammation to immunosuppression.

Macrophages are primary targets of lactate−mediated Kla in sepsis; however, their functional outcomes are highly context−dependent. On one hand, in a mouse model of septic cardiomyopathy, regular exercise enhances glycolysis in monocytes, promoting lactate production and subsequent H3K18la epigenetic modification. H3K18la facilitates the formation of an iNOS^+^Arg1^+^ macrophage subpopulation by modulating the expression of reparative genes such as Arg1, thereby accelerating the transition of cardiac macrophages from a pro−inflammatory to a reparative phenotype and restoring cardiac immune homeostasis (92). Additionally, disruption of the glycolysis–lactate–H3K18la axis impairs autophagy through transcriptional downregulation of Atg5 and Atg16l1, thereby compromising bactericidal activity in sepsis (113). Mechanistically, engagement of the FGF15/FGFR4 axis instigates the NF2–Hippo cascade, subsequently diminishing H3K18la-facilitated Irf7 transcription, thereby constraining M1 (114) macrophage polarization and attenuating systemic inflammatory insults (114). Beyond histone modifications, Kla of HMGB1 in macrophages—mediated by a p300/CBP-dependent mechanism—promotes HMGB1 secretion into the circulation. Secreted exosomal HMGB1 further disrupts endothelial integrity and increases vascular permeability, revealing a deleterious role of lactate in sepsis (73). These findings indicate that identical lactylation mechanisms can yield distinctly different functional outcomes depending on the substrate and cellular context.

Trained immunity and immune tolerance: Trained immunity—defined as the persistent functional reprogramming of innate immune cells following an initial stimulus—provides a robust theoretical framework for understanding the long-term immunological impact of lactate in sepsis. Previous studies have established that aerobic glycolysis induced via the Akt–mTOR–HIF-1α axis constitutes the metabolic basis of trained immunity, and that myeloid cell–specific HIF-1α deficiency abrogates the capacity to mount trained immunity against bacterial sepsis (41). Although that study primarily considered lactate as a byproduct of metabolic reprogramming rather than a signaling mediator, it revealed how sustained glycolytic enhancement epigenetically remodels immune cell function, thereby providing an upstream metabolic logic for subsequent elucidation of lactate−linked immune dysregulation via Kla in sepsis.

Neutrophil dysfunction and NETosis: In sepsis, neutrophils undergo substantial functional alterations via Kla. HMGB1 lactylation—occurring through a p300/CBP-dependent mechanism (73)—is associated with the formation of neutrophil extracellular traps (NETs). Specifically, lactate induces HMGB1 lactylation and its secretion from renal tubular epithelial cells; secreted HMGB1 then enters neutrophils and induces NET formation, thereby exacerbating sepsis-associated acute kidney injury (SA-AKI) (103). Circulating NET levels positively correlate with HMGB1 lactylation, and pre-administration of the HMGB1 inhibitor glycyrrhizic acid or the LDHA inhibitor oxamate reverses NET upregulation and attenuates AKI severity (103). Furthermore, HMGB1 siRNA effectively reduces lactate-mediated HMGB1 lactylation and alleviates AKI severity (103). This HMGB1–NETosis axis illustrates how Kla serves as a critical node connecting metabolic stress to neutrophil-mediated tissue injury.

T cells and adaptive immunity: Although the impact of lactate on adaptive immunity in sepsis is less well-studied than its effects on innate immunity, it is increasingly recognized. Lactate-induced suppression of T lymphocyte activation in sepsis is associated with altered expression of kinase-related genes. Elevated lactate levels downregulate CD40LG and SOCS3 expression, thereby inhibiting T-cell activation through suppression of the JAK–STAT signaling pathway (106). In the tumor field, elevated extracellular lactate promotes CD44 lactylation on CD8^+^ T cells, impairing hyaluronic acid binding and AKT signaling, and ultimately suppressing antitumor immunity (109). Similarly, in small cell lung cancer (SCLC), high lactate levels activate Nur77 expression in naïve CD8^+^T cells via H3K18la, enhancing TCR tonic signaling, suppressing T-cell function, and reducing immunotherapeutic efficacy (110). Furthermore, at the level of regulatory T cells (Tregs), lactate modulates the transcriptional activation of NF-κB p65 via H3K18la, subsequently upregulating TNFR2 expression, enhancing the immunosuppressive function of Tregs, and promoting the progression of malignant pleural effusion (MPE) (111). Despite these advances in other disease contexts, our mechanistic understanding of how Kla directly regulates T-cell function in sepsis remains limited.

Collectively, the current evidence is largely derived from associative studies or *in vitro* stimulation models, with a marked paucity of studies establishing causality through conditional gene knockout or site-specific mutagenesis. This represents a critical knowledge gap, particularly given the central role of T-cell dysfunction in sepsis-induced immunoparalysis and secondary infections. In summary, the immunological effects of lactate and Kla in sepsis exhibit remarkable cell type specificity and context dependency. In macrophages, H3K18la can exert either protective effects (promoting reparative polarization) or deleterious effects (driving M1 polarization and HMGB1 secretion). In neutrophils, HMGB1 lactylation propagates NETosis and organ injury. In T cells, lactate induces immunosuppression via JAK/STAT pathway inhibition. This functional diversity underscores that therapeutic strategies targeting lactate/Kla in sepsis must integrate considerations of cell type, disease stage, and the specific Kla substrates involved.

Notably, even within the same cell type—macrophages—H3K18la exerts dual functions depending on the specific substrate and pathological context (Arg1-driven repair versus HMGB1-driven injury). This intra-cellular duality, together with the inter-cell-type dichotomy described in parenchymal organs, underscores the complexity of context-dependent Kla regulation in sepsis.

## Discussion

This Review systematically integrates current evidence on the role of lactate and its induced lysine lactylation (Kla) in the pathophysiology of sepsis, delineating a progressive conceptual evolution in which lactate is increasingly recognized not merely as a metabolic waste product, but as a signaling intermediate and, more recently, as an epigenetic mediator linked to sepsis pathology. This shift not only reshapes our understanding of hyperlactatemia in sepsis but also opens new therapeutic dimensions for clinical practice. The following sections provide an in−depth discussion of the limitations and value of lactate as a biomarker, the dilemma of strategies targeting lactate metabolism, the pathological significance and therapeutic potential of lactylation modifications, and the key challenges of cell type specificity and precision delivery.

Although blood lactate level has been incorporated into the Sepsis−3 diagnostic criteria, the data cited in this Review indicate that the prognostic accuracy of a single lactate measurement falls far short of an ideal biomarker. This limitation arises from the dynamic and multifactorial nature of lactate levels: in sepsis, lactate elevation results from tissue hypoxia, inflammation−associated aerobic glycolysis, mitochondrial dysfunction, and impaired hepatic/renal function. Therefore, a single time−point measurement cannot distinguish among these different causes. In contrast, continuous monitoring of lactate clearance or trajectory slope provides greater clinical insight (29). From a pathophysiological perspective, a persistently increasing lactate trajectory reflects ongoing immunometabolic reprogramming (e.g., Warburg effect in macrophages and neutrophils) (38, 39)and progressive failure of clearance organs (56–58). Future clinical trials should therefore incorporate lactate clearance as a quantifiable intermediate endpoint to construct multidimensional prognostic models.

In therapeutic strategies targeting lactate metabolism and transport, inhibition of lactate production shows a double−edged effect. Studies have shown that the broad−spectrum LDH inhibitor oxamate and the specific LDHA inhibitor GNE−140 both effectively reduce intracellular lactate accumulation and histone lysine lactylation (e.g., H3K18la) levels in cardiomyocytes, and significantly alleviate hypertrophic phenotypes (115). However, the physiological functions of these enzymes are ubiquitous: LDH is present not only in the heart but also in immune cells, neurons (116), and erythrocytes (117). Therefore, systemic LDH inhibition may induce an energy crisis in normal tissues, especially in patients with pre−existing mitochondrial dysfunction in late−stage sepsis, where diverting pyruvate to mitochondrial oxidation could exacerbate oxidative stress and cell damage. Whilst dichloroacetate (DCA) has demonstrated encouraging chemosensitizing activity in preclinical models and early-phase clinical studies, its clinical translation is critically constrained by dose-limiting toxicities, most notably peripheral neuropathy and the attendant risk of demyelinating neurotoxicity. Consequently, a comprehensive and prudent evaluation of its safety profile in the oncological setting is imperative prior to broader clinical application (118) MCT1 inhibitors (e.g., AZD3965) have already entered phase I/II clinical trials in oncology, and preliminary studies have shown that they exhibit good tolerability and pharmacodynamic activity at target doses (119); however, their application in sepsis confronts interdisciplinary challenges. Selective inhibition of MCT4 could theoretically reduce extracellular lactate accumulation (120), thereby alleviating lactate−mediated immunosuppression. However, MCT1 in the central nervous system is responsible for lactate uptake as an alternative energy source (121), and systemic inhibition may lead to neurological deficits. Notably, in septic alveolar epithelial cells, downregulation of MCT1 expression itself exacerbates intracellular lactate accumulation and epithelial−mesenchymal transition, suggesting that restoring, rather than inhibiting, MCT1 may be more protective (55). These findings indicate that the tissue distribution and disease stage−specific expression patterns of MCT subtypes determine the complexity of intervention strategies. Future directions include the development of MCT4−selective inhibitors.

Pathological significance and therapeutic potential of lactylation modifications. The discovery of Kla is a landmark discovery in the field of lactate biology (71). This Review summarizes functional evidence for multiple histone and non−histone Kla sites in sepsis, as well as the validated and potential writers and erasers in sepsis (all summarized in tables), revealing the core driving role of lactylation modifications in organ injury. A remarkable illustration of context−dependent epigenetic regulation is that the same histone modification, H3K18la, exerts opposing biological outcomes across different cell types. In monocytes, H3K18la selectively enriches at the promoter of the repair−associated gene Arg1, driving macrophage polarization from a pro−inflammatory to a reparative phenotype and thereby effectively restoring cardiac immune homeostasis (92). In stark contrast, within the pulmonary niche, lactate elevates p300−mediated H3K18la occupancy at the METTL3 promoter, which enhances METTL3 transcription. This lactate−induced METTL3 upregulation, operating through a YTHDC1−dependent m^6^A modification axis, stabilizes ACSL4 mRNA, ultimately triggering ferroptosis in alveolar epithelial cells and exacerbating sepsis−associated lung injury (98)]. Similarly, in renal tubular epithelial cells, lactate−driven H3K18la activates RhoA transcription, which in turn initiates the RhoA−ROCK1 signaling cascade, promotes Ezrin phosphorylation, and culminates in NF−κB pathway activation, thus contributing to the pathogenesis and progression of sepsis−associated acute kidney injury (SA−AKI) (102). Admittedly, the deep−seated mechanisms underlying the differential targeting of the same histone modification site (H3K18la) across distinct cell types remain largely unexplored, and current evidence does not permit definitive mechanistic inference. In the interest of academic rigor, we have refrained from over−extrapolation and have instead designated this as a priority area for future investigation, calling for systematic studies that integrate cell-type-specific chromatin accessibility maps to elucidate the molecular logic from the perspective of epigenetic regulatory networks. Notably, indiscriminate systemic targeting of lactate metabolism or p300 acetyltransferase activity may non−specifically interfere with H3K18la−mediated compensatory reparative signals in macrophages, thereby undermining host immune protection. Therefore, future precision intervention strategies should aim to achieve an “uncoupling” of protective versus pathogenic Kla signaling—for example, by employing cell−type−specific promoter−driven CRISPR–dCas9 epigenetic editing systems to guide delactylases to specific pathogenic gene loci, or by designing ligand−modified lipid nanoparticles and other targeted delivery vehicles to selectively deliver epigenetic modulators to alveolar or tubular epithelial cells. Such approaches hold the promise of preserving the protective effects of H3K18la in immune cells while specifically blocking its pathogenic signaling in parenchymal cells, thereby opening a viable avenue for precision epigenetic therapy in sepsis.

Beyond histone lactylation, accumulating evidence has underscored the functional significance of non−histone Kla substrates in sepsis pathogenesis. For instance, Fis1−K20la has been shown to induce excessive mitochondrial fission and ATP depletion (101); Ezrin−K263la activates NF−κB signaling and promotes renal tubular epithelial cell apoptosis (102); and HADHA−K166/728la impairs fatty acid oxidation, thereby exacerbating septic cardiomyopathy (91). Collectively, these observations indicate that lysine lactylation exerts its effects beyond epigenetic regulation of gene transcription, extending to direct post−translational modification of metabolic enzymes and cytoskeletal proteins, thereby enabling rapid functional remodeling of cellular machinery. This multifaceted regulatory landscape echoes recent findings in the cancer field, where non−histone lactylation of PKM2, p53, and other substrates has been implicated in metabolic reprogramming and tumor progression (84). Notably, the first small−molecule inhibitor targeting the AARS2–AP−2γ protein–protein interaction, Kukoamine A, has been reported to exert synergistic antitumor effects when combined with PD−1 blockade (107). However, no such studies exist in sepsis models. This gap represents an important opportunity for future research, although the cell−type selectivity and safety profile of AARS1/2 inhibitors will require re−evaluation in the septic context.

Comparison with cancer research and implications. In recent years, research on lactylation in tumor biology has progressed rapidly (119). In the tumor microenvironment (TME), lactylation mainly promotes immune evasion (122, 123) and metabolic reprogramming (108); Unlike in cancer, lactylation in sepsis is characterized by a more widespread involvement of systemic and multiple organ damage (92, 101, 103). Specifically, lactate accumulation resulting from the Warburg effect in the TME induces histone and non−histone lactylation, thereby epigenetically reprogramming immune cells and promoting an immunosuppressive phenotype (112); whereas in sepsis, lactylation originates from the collective involvement of systemic immune cells (macrophages (104), neutrophils (103)) and parenchymal organs (lung (74), kidney (101), heart (92). Furthermore, histone lactylation in tumors can promote macrophage polarization toward the M2 phenotype and enhance their immunosuppressive phenotype, and lactylation may also regulate T cells, natural killer cells, dendritic cells, and myeloid−derived suppressor cells, thereby affecting anti−tumor immune responses (124). In late−stage sepsis, similar lactylation effects may exacerbate immune paralysis and the risk of secondary infection. Therefore, despite overlapping molecular mechanisms, the pathological context dictates distinctly different intervention strategies: inhibition of lactylation in cancer therapy can restore immune surveillance, whereas in sepsis, it may be necessary to enhance or inhibit lactylation in specific cell types in a stage−dependent manner.

The delivery of epigenetic modulators to septic patients with multiple organ dysfunction (MOD) presents four core challenges and corresponding translational countermeasures. Challenge 1: Dysregulated biodistribution. Capillary leakage, interstitial edema, and endothelial glycocalyx degradation cause non−specific extravasation of macromolecular agents, while dynamic changes in mononuclear phagocyte system (MPS) function—early hyperactivation and late immunoparalysis—lead to variable clearance rates of nanocarriers. Challenge 2: Hepatic and renal failure destabilize pharmacokinetics and toxicity. Impaired drug filtration, reabsorption, and degradation prolong half−life; risks of p300/CBP inhibitors (myelosuppression), HDAC inhibitors (thrombocytopenia), and AARS1−targeted agents (interference with tRNA aminoacylation and mitochondrial function) are magnified in energy−depleted MODS patients. Challenge 3: Hemodynamic instability and concomitant therapies. Vasopressors, continuous renal replacement therapy (CRRT), and extracorporeal membrane oxygenation (ECMO) may alter drug clearance via adsorption or ultrafiltration; acute−phase proteins (CRP/SAA) can non−specifically adsorb targeting ligands, compromising specificity. Challenge 4: Disease phase heterogeneity. The Kla landscape differs markedly between early hyperinflammation and late immunosuppression, and DAMPs (HMGB1, CIRP) induce sustained MPS uptake of carriers, interfering with the intended epigenetic reprogramming. To overcome these obstacles, we propose a “local delivery + ex vivo manipulation + dynamic monitoring” framework: intra−tracheal aerosolization for lung injury, renal artery perfusion or urinary exosome−based carriers for kidney injury, and adoptive transfer of ex vivo−modified monocytes to bypass systemic delivery barriers.

In contrast to previous reviews that have largely focused on the fundamental mechanisms of histone lactylation, the present work provides, for the first time, a systematic integration of the complete regulatory network of lactylation “writers” and “erasers” in the context of sepsis, encompassing the recently identified non−canonical lactyltransferase AARS1, and presents this information in two comprehensive summary tables. Furthermore, moving beyond the conventional emphasis on H3K18la, this review offers the first systematic depiction of the functional landscape of non−histone lactylation substrates across diverse septic organs and cell types (**Table 3**). Importantly, we are the first to explicitly delineate the functional duality of a single lactylation site (H3K18la) in distinct cell types, and based on this observation, we propose a framework for cell−type−selective intervention. Collectively, this systematic synthesis not only provides a clear regulatory network landscape for future in−depth mechanistic investigations of lactylation in sepsis, but also establishes a theoretical foundation for the development of precision intervention strategies guided by cell−type specificity.

## Outstanding questions and future directions

Most of the currently identified Kla writers (p300/CBP, KAT2B, AARS1) and erasers (SIRT1, SIRT3, HDAC2, HDAC9) are known acetylation modification enzymes, which facilitates drug repurposing. However, based on the current evidence, we are unable to conduct an in-depth and rational analysis of the dual and contradictory roles of the HDAC family as both lactylation “writers” and “erasers.” Therefore, we have compiled these fragmentary findings into summary tables, aiming to provide clearer and more convenient reference for future investigators working on the in-depth molecular mechanisms of HDAC family-mediated lactylation in sepsis.

There exists a hitherto unbridged “translational gap” between circulating lactate levels and tissue Kla deposition—no direct evidence supports a linear correlation between blood lactate concentration and Kla abundance in specific tissues. Indeed, Kla levels are governed by multiple factors, including intracellular lactate concentration, MCT−mediated transmembrane transport efficiency, local metabolic flux, and regional activities of Kla “writers” and “erasers,” with plasma lactate being only one of the upstream variables. To date, only Chu et al. (Front Immunol, 2022) have reported that H3K18la in PBMCs may serve as a diagnostic marker for septic shock (104); but that study did not simultaneously measure circulating lactate levels (104). To bridge this gap, we propose that future prospective cohort studies should concomitantly measure paired plasma lactate, global Kla and H3K18la levels in PBMCs, and evaluate through multivariate analysis whether Kla provides incremental prognostic value beyond lactate, SOFA, or qSOFA scores.

Second, according to the available literature, the AARS1/2−mediated non−coenzyme−A−dependent lactylation pathway primarily modifies non−histone metabolic enzymes and damage−associated molecular patterns (DAMPs), whereas the ACSS2–p300/CBP pathway is mainly responsible for histone Kla−mediated transcriptional reprogramming. However, the temporal contributions, substrate preferences, and functional crosstalk between these two pathways during the progression of sepsis have not been systematically investigated—representing a highly promising area of exploration. To address this question, we propose generating myeloid−specific conditional knockout mice (Acss2^fl/fl^ and Aars1^fl/fl^) and, following LPS or CLP model induction, collecting tissues including liver, spleen, lung, kidney, and immune cells for global lactyl−proteomic profiling to systematically delineate the division of labor between the two pathways in terms of temporal dynamics and substrate selection. If validated, this model would provide a theoretical basis for devising precision intervention strategies tailored to different stages of sepsis.

Finally, regarding the crosstalk between lactylation and other PTMs, we hypothesize that under conditions of lactate surge in sepsis, Kla competes with acetylation and ubiquitination in a “temporally biased” manner at specific lysine residues (e.g., HMGB1−K42, p53−K120), thereby altering the nucleocytoplasmic translocation, stability, and downstream signaling output of substrate proteins. To test this hypothesis, sequential immunoprecipitation coupled with targeted mass spectrometry should be employed to quantify the relative occupancy of different modifications on the same peptide, and unnatural amino acid mutagenesis should be used to generate “Kla−only” mutants to directly assess their functional effects. If Kla is confirmed to act as a “PTM switch” regulating the binary functional outcomes of key signaling proteins, it would provide a theoretical foundation for combination therapeutic strategies jointly targeting “writers” and “erasers.”

The three directions outlined above are not mutually exclusive—the compartmentalized distribution of tissue Kla depends on the temporal contributions of the two pathways, and the substrate preferences of these pathways directly influence the PTM competition landscape at specific residues. We advocate for the establishment of an integrated multi−omics analysis platform in sepsis mouse models (encompassing lactylome, acetylome, phosphoproteome, and ubiquitinome, combined with single−cell H3K18la epigenomics) to systematically address these questions within the same set of samples. Such studies are expected to provide new therapeutic perspectives for precision epigenetic intervention in sepsis, stratified by disease stage, cell type, and modification site.
